# Abdominal-based vs. alternative flaps: Surgical outcomes and quality of life following different techniques of autologous breast reconstruction

**DOI:** 10.1016/j.jpra.2025.12.011

**Published:** 2025-12-20

**Authors:** Lisanne Grünherz, Stella Vocke, Laura C. Siegwart, Victoria Wlach, Anna Burger, Pietro Giovanoli, Nicole Lindenblatt, Duveken B.Y. Fontein

**Affiliations:** a Department of Plastic Surgery and Hand Surgery, University Hospital Zurich, Zurich, Switzerland; b Department of Plastic, Reconstructive, Aesthetic, Hand- and Burn Surgery, Munich Clinic Bogenhausen, Technical University Munich, Munich, Germany; c TUM School of Medicine and Health, Technical University Munich, Munich, Germany; d Department of Hand, Plastic and Reconstructive Surgery, Microsurgery, Burn Center, BG Trauma Center Ludwigshafen, Hand and Plastic Surgery, University of Heidelberg, Heidelberg, Germany; e Department of Traumatology, University Hospital Zurich, Zurich, Switzerland

**Keywords:** Autologous breast reconstruction, Quality of life, Deep inferior epigastric artery perforator flap, Breast cancer, Mastectomy, Quality of care

## Abstract

Abdominal-based flaps are considered the gold standard for autologous breast reconstruction (ABR). With the increasing number of women undergoing mastectomy, however, alternative free flaps from other regions have emerged as valuable alternatives when patients do not qualify for an abdominal-based flap. This single-center cohort study evaluates objective and subjective outcomes in patients who underwent abdominal-based versus alternative flaps for breast reconstruction.

Patients who underwent ABR between 2010 and 2022 were included. Baseline patient characteristics and data on oncological and surgical treatment were collected. Since 2018, all patients received the validated BREAST-Q questionnaire evaluating satisfaction and quality of life preoperatively and at regular intervals following surgery. Multinomial logistic regression evaluated the probability of flap complications based on the type of flap. We also evaluated quality of life between abdominal-based and alternative flaps.

Of the 183 patients were included in this study (146 abdominal-based and 37 alternative flaps). We could not ascertain a relationship between type of flap and flap complications. More donor-site complications and more secondary aesthetic corrective surgery were noted in patients who underwent abdominal-based flaps. Our results did not show any differences between patients who underwent abdominal-based compared to alternative flap types for various satisfaction domains of the BREAST-Q.

Abdominal-based and alternative free flap types are both viable treatment options in ABR and several variables need to be considered when selecting the appropriate flap for the right patient. Careful flap selection and the shared decision-making process between patient and surgeon reflect high patient-satisfaction and an optimal aesthetic result.

## Introduction

Microsurgical, free flap Autologous breast reconstruction (ABR) has emerged as a very reliable option for women after mastectomy without the risk of implant-associated complications. Further advantages include a natural breast shape and feel with the unique chance to combine optimized shaping and sizing of the breast with a body contouring donor site procedure.^1^, ^2^, ^3^, ^4^ A growing body of literature has also demonstrated its positive impact on quality of life and psychosocial well-being.^5^

Since it was first described by Koshima in 1989, the deep inferior epigastric perforator (DIEP) flap has become the gold standard for ABR.^6^ Unlike traditional methods that are based on the use of the rectus abdominis muscle, the DIEP flap preserves the abdominal muscles, minimizing postoperative complications and promoting a faster recovery. A further refinement in the transfer of lower abdominal tissue is the superficial inferior epigastric artery (SIEA) flap that does not require any incision of the rectus abdominis fascia or muscle leaving these structures completely untouched.^7^

Considering the increasing number of women undergoing either therapeutic or risk-reducing mastectomies and subsequent reconstruction, alternative free flaps from the thigh, buttock or lower back such as the profunda artery perforator flap (PAP), transverse myocutaneous gracilis (TMG), or the superior gluteal artery perforator flap (SGAP) flap have emerged as valuable alternatives for women with insufficient abdominal donor-site tissue.^8^^,^^9^ In recent years, a 31 % increase in perforator flaps from the thigh and lower back has been reported for ABR.^10^ While the DIEP flap still remains the gold standard, the most suitable flap is based on an individual's anatomy as well as patient’s and surgeon’s preferences. The objective of this study was to evaluate surgical outcomes and patient satisfaction and quality of life in patients who underwent ABR with abdominal-based flaps versus alternative flap types between 2010 and 2022.

## Material and methods

### Patient collective

We conducted a single center observational study on all patients who underwent ABR at the Department of Plastic Surgery and Hand Surgery between 2010 and 2022 at the University Hospital of Zürich, Switzerland. Ethical approval was given by the Cantonal Ethics Committee of Zurich, Switzerland (Ethical approval Nr.: 2021-01591 and 2018-02058). Written informed consent was obtained from all patients. Since 2018, all patients received the BREAST-Q preoperatively as well as at 6 weeks, 6 months, 12 months, and 24 months postoperatively. Exclusion criteria for the BREAST-Q included patients who were not able to complete questionnaires due to insufficient knowledge of the German or English language, impaired psycho-intellectual abilities or any psychiatric disorder. These patients were only included for an analysis of their patient characteristics and surgery details, provided written informed consent for the use of data was given.

Medical records were analyzed with respect to patients characteristics including age, body mass index (BMI), current history of smoking, reason for mastectomy, genetic predisposition to breast cancer, irradiation, chemotherapy, history of abdominal operations, concomitant diseases, timing for breast reconstruction, flap technique, length of surgery, flap weight, flap and donor-site complications as well as refinement operations.

### Statistical analysis

Statistical analysis of patients’ baseline characteristics was performed using descriptive and summary statistics to identify a central tendency. Data was analyzed using IBM SPSS Statistics Version 29 (IBM, Armonk, NY, USA), Microsoft® Excel Version 14.3.6. (Microsoft Corp., Redmond, WA, USA) and GraphPad Prism Version 7.04 (GraphPad, La Jolla, CA, USA). We performed binary and multinomial logistic regression analyses (confidence intervals [CI] were set at 95 %, *p*-values <0.05 were considered statistically significant) to assess the likelihood of flap and/or donor-site complications in relation to flap type. We adjusted our analyses for the following confounders: obesity, smoking and prior radiotherapy. Patients with missing data for specific variables were excluded from analyses requiring those specific variables.

Quality of life was assessed using the BREAST-Q® (German Version).^11^ The content and design of the questionnaire are protected by U.S. and international intellectual property laws, and use was made under a license from Memorial Sloan Kettering Cancer Center, New York, NY, USA. For further information on permitted use. Data from the BREAST-Q® questionnaires were evaluated using the designated software program Q-Score (Copyright © 2012, Memorial Sloan Kettering Cancer Center and the University of British Columbia).^12^ Measures were linearly converted to a 0–100 scale. Differences in BREAST-Q outcomes between patients who underwent abdominal-based flaps and alternative flaps were analyzed using independent samples T-tests for normally distributed data and the Mann-Whitney U Test for non-normally distributed data. Fisher’s exact test was performed to compare two categorical variables and Chi-square test for three or more categorical variables. Confidence intervals were set at 95 %. A *p*-value of <0.05 was considered statistically significant. The manuscript was checked against the Strengthening the Reporting of Observational Studies in Epidemiology (STROBE) checklist (supplemental Appendix: STROBE Checklist).

## Results

**Figure 1:**
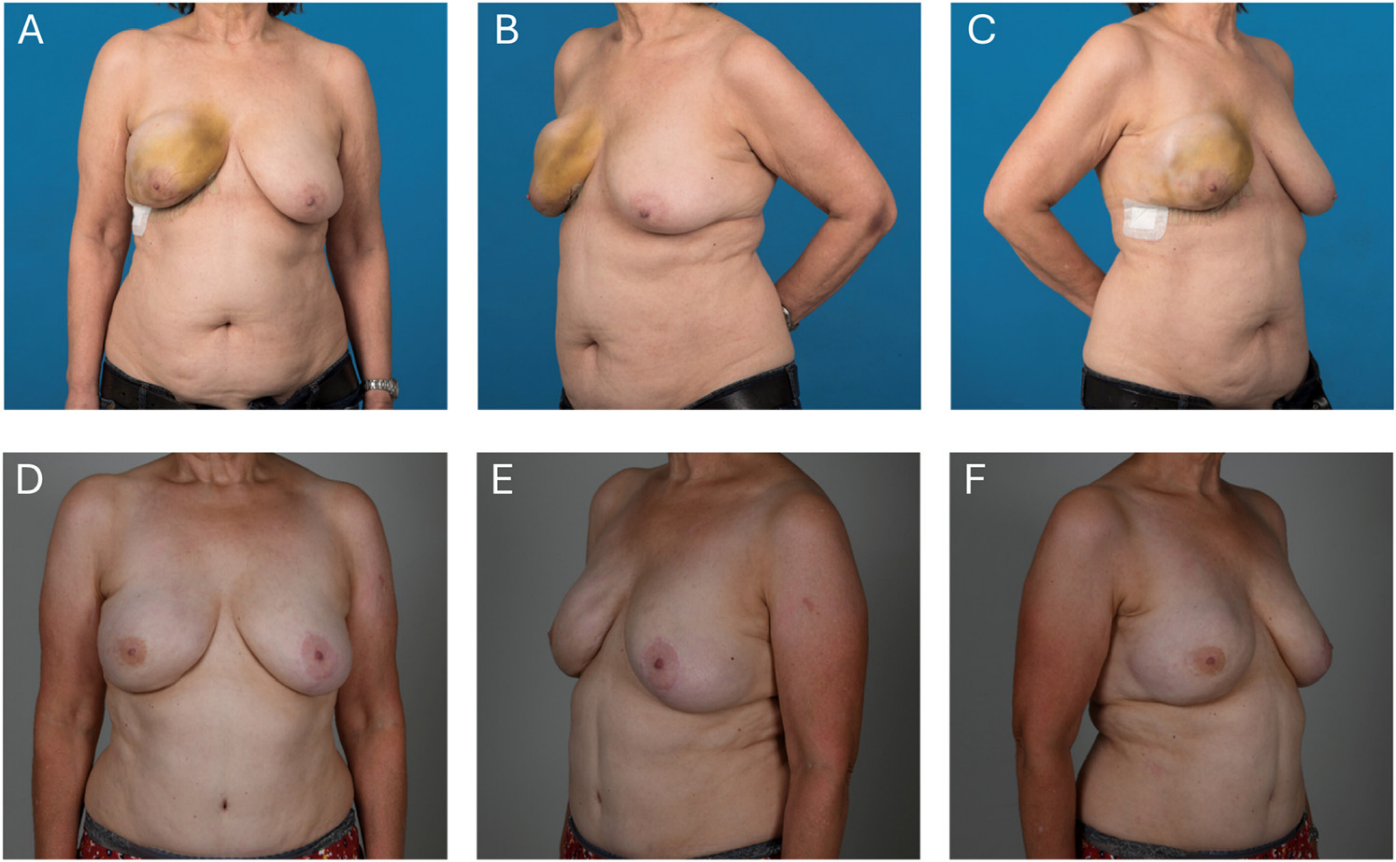
(A-C) Preoperative photographs of a 62-year-old woman with a local recurrence of a breast carcinoma to the right breast. (D-F) 16 months after right-sided nipple-sparing mastectomy plus autologous breast reconstruction with a DIEP flap. Refinements included removal of the skin island of the DIEP flap in the inframammary fold and a left sided mastopexy.

**Figure 2:**
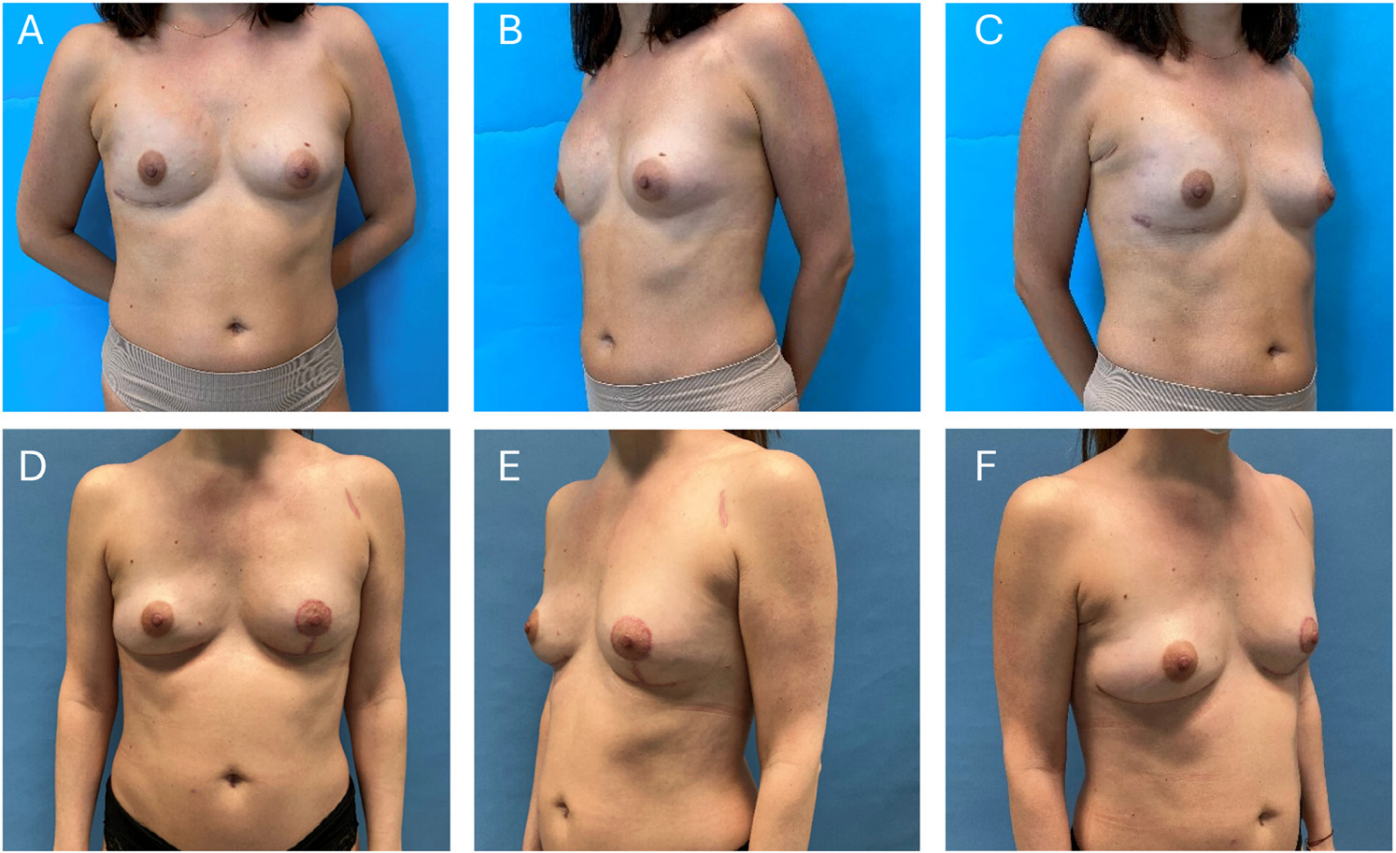
(A-C) Preoperative pictures of a 37-year-old woman with capsular contracture after immediate implant-based reconstruction after nipple-sparing mastectomy of her right breast. (D-F) 12 months after autologous breast reconstruction with a PAP flap and mastopexy of the left breast. During the further course the skin island of the PAP flap, located in the submammary fold, was removed. At the same time bilateral lipofilling was performed.

We included 183 patients who underwent ABR between 2010 and 2022. Baseline characteristics of patients included in this study are depicted in Table 1. Of the 146 patients received an abdominal-based free flap, while in 37 patients the free flap was harvested from alternative donor-sites (thigh, gluteal, others). Both immediate and delayed reconstructions (122 and 98 respectively) as well as unilateral and bilateral (146 and 37 respectively) reconstructions were included in this study. In this study, abdominal-based flaps included DIEP (*n* = 152), MS-TRAM (*n* = 20), SIEA (*n* = 5), and TRAM (*n* = 1) flaps, whilst alternative flap types consisted of TMG (*n* = 19), PAP (*n* = 15), SGAP (*n* = 2), IGAP (*n* = 1), and PMT (*n* = 5). Figure 1 depicts an abdominal-based flap type (DIEP) and Figure 2 an alternative flap (PAP-flap) type. Mean age of patients included in this study was 49.3 years (SD 9.1) for abdominal-based flaps and 47.1 years (SD 11.1) for alternative flap types and did not show a statistically significant difference. Descriptive analysis of breast cancer characteristics, including primary or recurrent disease, therapeutic or risk-reducing mastectomy and genetic predisposition showed an equal distribution between the groups. There was also no difference regarding relevant risk factors. As expected, a lower BMI was noted in patients who received an alternative free flap (22.2 kg/m^2^ ± 3.3 vs. 25.6 kg/m^2^ ± 3.6, *p* < 0.0001). In most patients (*N* = 122) an immediate ABR was performed in a two-team approach with the gynecologist. The alternative flaps also had a significantly lower flap weight when compared with the abdominal-based group (320 g ± 160 g vs. 585 g ± 222 g, *p* < 0.0001).

**Table 1:** Baseline characteristics of patients included in this study.

Variable	Abdominal-based		Alternative		*p*-value
	N	%	N	%	
Patients	146		37		
Flaps	178		42		
TRAM	1	<1			
MS-TRAM	20	11			
DIEP	152	85			
SIEA	5	3			
TMG			19	45	
PAP			15	35	
SGAP			2	5	
IGAP			1	2	
PMT			5	12	
Age [years], *mean (SD)*	*49.3*	*9.10*	*47.1*	*11.10*	0.20
Age [years]					
<20	0	0	0	0	
20–40	17	12	11	30	
40–60	113	77	22	59	
>60	16	11	4	11	
BMI [kg/m^2^], *mean (SD)*	*25.6*	*3.6*	*22.2*	*3.3*	0.0001
BMI [kg/m^2^]					
15–19.9	3	2	8	21	
20–24.9	62	42	25	68	
25–29.9	58	40	3	8	
30–34.9	22	15	0	0	
≥35	1	<1	1	3	
Breast cancer					
Primary disease	119	82	30	86	
Recurrent disease	21	14	5	14	
Risk factors					
Diabetes mellitus	4	3	1	3	0.9
Obesity (BMI ≥ 30 kg/m^2^)	23	16	1	3	0.04
Active smoker	32	22	10	27	0.52
Preoperative chemotherapy	76	52	17	46	0.58
Preoperative radiotherapy	55	37	12	32	0.7
Flaps	178		42		
Unilateral	114	78	32	86	0.36
Bilateral	32	22	5	14	
Breast reconstruction					
Immediate	98	55	24	57	0.86
Delayed	80	45	18	43	
Flap weight [g], *mean (SD)*	*585*	*212*	*320*	*160*	0.0001
Operation time [min], *mean (SD)*					
Unilateral	*392*	*102*	*366*	*90*	0.19
Bilateral	*572*	*219*	*612*	*114*	0.36

BMI, body mass index; SD, standard deviation.

### Flap complications

Seven abdominal-based flaps and one alternative flap suffered partial necrosis. Complete flap loss was noted in 5 patients (2.7 %) (three abdominal-based (2 %) and two other flap types (5.4 %), *p* = 0.469). We performed both adjusted and unadjusted multinomial logistic regression analyses to evaluate the probability of flap complications (partial or complete flap loss) based on the type of flap used. No relationship between the type of free flap and the odds of flap complications (partial or complete flap loss) could be ascertained. Unadjusted analyses revealed an odds ratio of 1.711 (95 % CI: 0.203–14.393, *p* = 0.621) for partial flap loss in abdominal-based flaps and 0.367 (95 % CI: 0.059–2.284, *p* = 0.282) for complete flap loss. Correcting for possible confounders (preoperative radiotherapy, obesity, smoking status and timing of reconstruction) did not alter the results (Table 2).

**Table 2:** Odds ratios of flap complications and revisional surgery in patients who underwent abdominal-based versus other free flaps.

Variable	Partial flap loss (*n* = 8)			Complete flap loss (*n* = 5)			Complications requiring revisional surgery (*n* = 35)		
	Odds Ratio	Confidence Interval (95 %)	*p*-value	Odds Ratio	Confidence Interval (95 %)	*p*-value	Odds Ratio	Confidence Interval (95 %)	*p*-value
Unadjusted analyses									
Flap type									
abdominal-based (ref)	1.00			1.00			1.00		
other free flaps	1.71	0.203–14.393	0.621	0.37	0.059–2.284	0.282	0.196	0.045–0.857	0.030
Adjusted analyses									
Flap type									
abdominal-based (ref)	1.00			1.00			1.00		
other free flaps	1.78	0.203–15.548	0.603	0.29	0.041–2.059	0.22	0.226	0.051–1.004	0.051
Timing									
Immediate (ref)	1.00			1.00			1.00		
Delayed	2.50	0.407–15.377	0.322	1.52	0.231–9.943	0.67	0.794	0.331–1.901	0.604
Prior radiotherapy									
No (ref)	1.00			1.00			1.00		
Yes	2.02	0.390–10.504	0.401	n/a	n/a	n/a	1.514	0.620–3.695	0.363
Obesity									
No (ref)	1.00			1.00			1.00		
Yes	0.78	0.085–7.089	0.822	3.35	0.304–36.856	0.32	2.064	0.769–5.536	0.150
Smoking status									
Non-smoker (ref)	1.00			1.00			1.00		
Smoker	1.18	0.221–6.333	0.845	2.87	0.423–19.425	0.28	0.768	0.287–2.059	0.60

n/a, not applicable.

### Donor-site morbidity

Of the 33 patients (22.6 %) who underwent abdominal-based free flaps and 2 patients (5.4 %) who had alternative flaps required surgical revision of the donor site (*p* = 0.018). Most common donor-site complications for abdominal-based flaps included seroma (*n* = 15), bulging (*n* = 3), and wound-breakdown (*n* = 2). Logistic regression analyses were performed to assess the likelihood of donor-site complications requiring revision surgery based on the type of flap used. Abdominal-based flaps were associated with more donor-site complications requiring surgical revision (OR 0.196 (95 % CI 0.045–0.857, *p* = 0.03) when compared to alternative flaps. After adjusting for possible confounders, the results just did not reach statistical significance. None of the possible confounders showed a statistically significant effect on the risk of donor-site complications (Table 2).

### Refinement surgery

The analysis of touch-up procedures showed that 45.2 % of patients (*n* = 66) in the abdominal-based free flap group and 45.9 % of patients (*n* = 17) in the alternative free flap group received additional lipofilling of the reconstructed breast (*p* = 0.94) (Supplementary Table 1). Regarding other flap types, both TMG und PAP flaps showed similar numbers of lipofilling sessions (TMG vs PAP: 9 (56.3 %) vs. 6 patients (46.2 %), Pearson chi-squared *p* = 0.588). The number of sessions ranged from 1 to 4 with a mean of 1.5 sessions in both groups. Other touch-up procedures, including nipple reconstruction, scar correction and liposuction, were slightly more common in patients after an abdominal-based free flap breast reconstruction. Symmetrization surgery of the contralateral breast was performed in 51 abdominal-based flap patients (36.1 %) and in 11 (33.4 %) alternative flap patients (*p* = 0.759). Regarding donor-sites, we observed more aesthetic corrections of the abdominal donor-site than other donor sites (26.7 % vs. 5.4 %, *p* = 0.004).

### Patient-reported outcome measures

BREAST-Q Questionnaires were sent preoperatively and at regular intervals after the surgery. Twenty-one patients completed at least one postoperative BREAST-Q Questionnaire (13 abdominal-based flaps and eight alternative flaps). Our results did not show any differences in the domains ‘satisfaction with outcomes’ and ‘satisfaction with breasts’ between patients who underwent abdominal-based breast reconstruction and patients who had alternative flaps. Well-being domains (psychosocial, sexual, and physical) also did not show differences in scores between the abovementioned groups (Table 3). Patient numbers were too small to evaluate differences between subgroups of patients.

**Table 3:** Satisfaction and quality of life results in patients with abdominal versus other free flap types.

Adjusted analyses	Abdominal-based (*n* = 13)		Other free flaps (*n* = 10)		*p*-value
	mean	SD	mean	SD	
Satisfaction with breasts	63.31	19.00	69.25	15.11	0.463*
Satisfaction with outcome	71.83	23.19	80.50	15.32	0.366*
Satisfaction with nipples	60.44	35.25	43,00	37,32	0.48*
Psychological well-being	77.23	22.69	79.25	21.69	0.916⁎⁎
physical well-being (chest)	69.23	13.48	75.13	15.92	0.50⁎⁎
Sexual well-being	54.64	24.91	63.57	23.74	0.425⁎⁎
Satisfaction with information	67.31	21.31	72.00	17.12	0.456⁎⁎
Satisfaction with surgeon	81.62	18.04	92.63	14.09	0.185⁎⁎

SD, standard deviation.

⁎ independent samples T-Test.

⁎⁎ Mann-Whitney U Test.

## Discussion

This study focuses on differences in surgical and quality-of-life outcomes in patients who underwent abdominal-based versus alternative flaps for ABR. Flap complications were similar for both abdominal-based and alternative flap types, although donor-site morbidity was higher in abdominal-based flaps. Patient-satisfaction, however, did not differ based on the type of free flap used.

In this study, we aimed to evaluate the BREAST-Q outcomes between patients based on flap type. Our results showed similar levels of patient-satisfaction and well-being throughout various domains of the BREAST-Q. Especially with respect to psychosocial and sexual well-being, our findings reflect that both abdominal-based and alternative flap types are acceptable options that can meet patients’ expectations. This finding likely reflects optimal patient selection and individualized surgical planning. The appropriate flap type should be chosen based on a patient’s body shape and composition, donor site availability, patient preferences and overall reconstructive goals.^10^^,^^13^^,^^14^ Consequently, when a patient’s functional and aesthetic needs are met, both abdominal-based and alternative flap types, when properly selected and executed, can result in similarly high satisfaction levels across all domains addressed by the BREAST-Q. Although not all patients completed a 24-month follow-up questionnaire, the time after surgery was long enough for complete healing and adaptation, during which patients were able to adapt to their reconstruction outcomes and long-term benefits to become apparent, regardless of flap type.

We observed a higher number of patients undergoing surgical revision of the abdominal donor-site. This is in line with Weitgasser et al. who found an increased donor-site morbidity of 23 % after double DIEP flap harvest compared with 16 % after double TMG for ABR.^15^ In contrast to the aforementioned study, however, other authors reported a significantly lower rate of abdominal donor-site complications in comparison with the thigh after PAP flap harvest.^16^^,^^17^ Aesthetic correction of the donor-site, however, was almost twice as high as after PAP flap harvest.^16^ Although, focus on improving the donor-site after DIEP flap harvest has steadily grown, aesthetically unpleasant scars as well as abdominal wall weakness are still observed in up to 30 % of the patients.^15^^,^^18^, ^19^, ^20^, ^21^ In 2020, we proposed an algorithm to reduce donor site morbidity and improve the aesthetic appearance of the abdominal scar, including meticulous donor site closure and careful perforator selection based on preoperative CT angiography and surgical evaluation.^21^ We believe that proper patient selection, considering patients with a normal to slightly elevated BMI, a local fat deposition in the lower abdomen and abdominal skin redundancy for DIEP flap harvest, are important aspects to optimize donor-site morbidity.

Women with a remarkably lower BMI tend to undergo ABR with an alternative free flap.^22^^,^^23^ Not surprisingly, this results in a lower overall flap weight, which is reflected in our results and was also observed in other studies.^15^^,^^16^ In this context, Weichman et al. showed that this flap weight was sufficient to provide body-appropriate breast volume in the low BMI patient collective.^22^

Interestingly, there was no difference in the number of touch-up procedures, particularly number of lipofilling sessions after using an abdominal-based versus alternative free flap for ABR. To the best of our knowledge, we only found one study comparing the number of touch-up procedures after different techniques of ABR. In contrast to our results, they reported a higher number of lipofilling sessions after ABR with TMG flaps compared with DIEP flaps, which might be attributed atrophy of the gracilis muscle and consecutive shrinkage of the flap.^15^ Furthermore, the choice of free flap is determined not only by available donor tissue and body composition, but also by the inherent advantages of different flap types. The DIEP flap’s versatility in inset techniques allows surgeons to optimize aesthetic outcomes through various shaping methods.^1^^,^^2^^,^^24^^,^^25^ Similarly, other flap types offer their own unique advantages and can achieve comparable outcomes when chosen for the appropriate patient, suggesting that the comparable numbers of refinement procedures reflect appropriate flap selection rather than limitations in any particular technique.

The results of our study need to be contextualized with the limitations arising from our relatively small sample size of patients who received alternative free flaps. In our study, 146 patients received an abdominal-based flan versus 37 alternative flap types. Consequently, the higher use of abdominal-based flaps may bias the comparative power of complication rates and PROMs, especially as response-rates to the questionnaires in this study were relatively low. For this reason, the results of the BREAST-Q are exploratory at most. A strength of this study, however, is the prospectively collected BREAST-Q, which was sent to patients preoperatively as well as postoperatively at various time intervals after surgery, potentiating more generalizable results once longer follow-up becomes available.

To conclude, although the DIEP flap is commonly referred to as the gold standard for breast reconstruction, both abdominal-based and alternative free flap types are viable treatment options in ABR and several variables need to be taken into account when selecting the appropriate free flap. The choice of free flap should ideally take into account multiple other factors, such as patient-preference, surgeon preference and experience, body composition and the availability of an acceptable donor-site as well as potential contraindications for an abdominal-based flap.^10^^,^^13^^,^^26^^,^^27^ Patients who are involved in a shared decision-making process are more likely to have better health outcomes and more satisfaction with their care.^28^^,^^29^ Understanding and discussing patient-preferences, expectations and surgical treatment options is imperative and plays a significant role in treatment decisions.^30^ Our findings underline the importance of integrating shared decision-making between patient and surgeon into the choice of flap type, aiming to provide more personalized, high-quality patient care.

## Funding

This research did not receive any specific grant from funding agencies in the public, commercial, or not-for-profit sectors.

## Ethical approval

Ethical approval was given by the Cantonal Ethics Committee of Zurich, Switzerland (Ethical approval No.: 2021-01591 and 2018-02058).

## Declaration of competing interest

Nicole Lindenblatt acts as consultant and scientific advisor for Medical Microinstruments (MMI). All other authors have no financial interests/personal relationships to declare.

## Appendix Supplementary materials

Image, application 1
