# Supplementary material for: Abdominal-based vs. alternative flaps: Surgical outcomes and quality of life following different techniques of autologous breast reconstruction
Source: JPRAS Open. 2025 Dec 20;48:644–53. doi: 10.1016/j.jpra.2025.12.011 (PMC12830217; doi:10.1016/j.jpra.2025.12.011)
Supplement: Supplementary file 1 [file mmc1.docx]

**Supplementary Table 1: Additional refinement surgeries in abdominal-based and alternative flap types**

|  |  | **Abdominal-based** | |  | **Other free flaps** | |  |
| --- | --- | --- | --- | --- | --- | --- | --- |
| **Procedure** |  | **N** | **%** |  | **N** | **%** | **p-value** |
| **Lipofilling** |  | 66 | 45.2 |  | 17 | 45.9 | 0.94 |
| number of sessions, *mean (range)* |  | *1.5* | *1-4* |  | *1.5* | *1-3* |  |
| **Other breast touch-up procedures** |  |  |  |  |  |  |  |
| nipple reconstruction |  | 60 | 34 |  | 7 | 17 |  |
| scar correction |  | 23 | 13 |  | 2 | 5 | 0.75 |
| liposuction |  | 24 | 14 |  | 1 | 2 |  |
| **Symmetrization of the contralateral breast** | |  |  |  |  |  |  |
| immediate |  | 35 | 24 |  | 5 | 14 | 0.18 |
| delayed |  | 16 | 11 |  | 6 | 16 |  |
| **Aesthetic correction of the donor-site** |  | 44 | 30 |  | 5 | 14 | 0.06 |
| scar correction |  | 16 | 11 |  | 1 | 2 |  |
| dog-ear excision |  | 20 | 14 |  | 3 | 7 |  |
| liposuction |  | 6 | 4 |  | 1 | 2 |  |
| correction of umbilicus |  | 2 | 1 |  | n/a | n/a |  |
| *n/a: not applicable* |  |  |  |  |  |  |  |
